# Linezolid induced psychosis and hallucination: Case report and literature review

**DOI:** 10.1016/j.amsu.2022.104654

**Published:** 2022-10-13

**Authors:** Hamza Maqsood, Salman Sajjad, Saba Aslam, Shifa Younus, Sadiq Naveed

**Affiliations:** a Nishtar Medical University and Hospital, Multan, Pakistan; b Child and Adolescent Inpatient Units, Institute of Living, Hartford, CT, USA; c University of Connecticut, Farmington, CT, USA; d School of Medicine at Quinnipiac University, North Haven, CT, USA

**Keywords:** Linezolid, Infection, Psychosis, Neuropsychiatric, Hallucinations, Confusion, Adverse drug reaction, Antibiotic

## Abstract

**Introduction:**

Various classes of antibiotics have been linked to causing a wide range of neuropsychiatric symptoms. These manifestations range from psychosis and delirium to encephalitis and intracranial hypertension. The prevalence of psychosis adverse drug reactions (ADRs) for individual antibiotics ranges from 0.3 to 3.8%. We report a rare case of linezolid-induced psychosis and hallucination.

**Case presentation:**

We report a 52-year-old Asian gentleman who presented with an altered level of consciousness and hallucinations. He was treated for third-degree burns of 31% of the body for two months. Based on clinical and laboratory investigations, linezolid-induced psychosis and hallucination were diagnosed. His Naranjo probability score was +8. The drug was stopped, and the patient recovered successfully.

**Conclusion:**

On rare occasions, toxic blood levels of linezolid can lead to neuropsychiatric manifestations. Both linezolid-induced psychosis and hallucinations are manageable by suspension of the drug. Therefore, physicians should monitor the blood levels of this antibiotic to keep their patients safe from such serious adverse effects.

## Highlights

• Various classes of antibiotics have been linked to causing a wide range of neuropsychiatric symptoms. These manifestations range from psychosis and delirium to encephalitis and intracranial hypertension.
• On rare occasions, toxic blood levels of linezolid can lead to neuropsychiatric manifestations.
• Both linezolid-induced psychosis and hallucinations are manageable by suspension of the drug. Therefore, physicians should monitor the blood levels of this antibiotic to keep their patients safe from such serious adverse effects.

## List of abbreviations

ADR: Adverse drug reaction
MRSA: Methicillin-resistant staphylococcal aureus
SSRIs: Selective serotonin reuptake inhibitors
SNRIs: Serotonin-norepinephrine reuptake inhibitor
RT-PCR: Reverse transcription-Polymerase chain reaction
DSM-5: Fifth edition of Diagnostic and Statistical Manual of Mental Disorders
GCS: Glasgow coma scale

## 1 Background

Various classes of antibiotics have been linked to causing a wide range of neuropsychiatric symptoms. These manifestations range from psychosis and delirium to encephalitis and intracranial hypertension [1]. It is well supported by the existing literature that there is a direct relation between the appearance of psychosis and hallucination and antibiotics exposure [2]. The presentation, severity, and prognosis of these neuropsychiatric manifestations depend upon the class of the antibiotics. The most well-established underlying pathophysiology includes the direct effect of antibiotics on neurotransmitters and their receptors and anti-inflammatory effects that may modify cytokine production, leading to the modulation of neurotransmitter function [3]. The most common antibiotics leading to the development of psychosis are penicillin, fluoroquinolones, cephalosporins, and anti-tuberculous drugs [4]. The prevalence of psychosis adverse drug reactions (ADRs) for individual antibiotics ranges from 0.3 to 3.8% [3]. Linezolid belongs to a synthetic class of antibiotics known as oxazolidinones with activity against many important bacteria, including methicillin-resistant staphylococcus and streptococcus [1]. We report the first case of a middle-aged gentleman who developed altered mental status, psychosis and auditory/visual hallucinations due to prolonged therapy with linezolid. Such cases add to the existing literature about the neuropsychiatric adverse effects of linezolid. Our case report is compliant with the SCARE Guidelines 2020 [5].

## 2 Case presentation

A 52-year-old gentleman presented to the emergency department of our hospital with an altered level of consciousness and hallucinations. Two months back, he was taken to the burn unit of the affiliated hospital, where he received treatment for a third-degree burn of 31% of the body. Two sets of blood cultures were done, which showed the growth of methicillin-resistant staphylococcal aureus (MRSA), Escherichia coli (E Coli), and Enterococcus fecalis (E fecalis) sensitive to linezolid. Intravenous ceftriaxone 1g and linezolid 600mg were started twice per day along with fluid and electrolytes resuscitation. He was managed there for a period of one month. His discharge medications included 600mg linezolid to be taken orally twice per day, multivitamins, topical antimicrobial ointment, and lavender oil.

On the day of presentation to the emergency department, the pulse rate was 84 beats/min, blood pressure was 115/70 mmHg, respiratory rate was 20/min, and oxygen saturation was 97%. As the patient was unable to give any history, the patient's attendants were interviewed. He was speaking incomprehensible words and had auditory and visual hallucinations. He complained of hearing whistling sounds and seeing some unknown persons who were not physically present there. He had no history of body weakness, seizures, or visual changes. Past medical history was insignificant except for third-degree burns of 31% of the body, for which he was taking linezolid 600mg twice per day from two months. Family history was negative for any neuropsychiatric disorder. He was not taking any other neurotropic medication like selective serotonin reuptake inhibitors (SSRIs), serotonin-norepinephrine reuptake inhibitors (SNRIs), monoamine oxidase inhibitors (MAOIs), or any other drug leading to manifestations of serotonin syndrome. The patient was non-alcoholic, non-smoker, and never abused drugs.

On general physical examination, the patient was not oriented in time and space and to person. His Glasgow coma scale (GCS) level was 9/15 (E3V2M4). There was no photophobia, neck stiffness, or facial asymmetry. The cranial nerves were intact. The patient was flexing to withdraw from painful stimuli on motor system examination. The rest of the CNS and systemic examination was normal. As per management protocol, a venous sample was drawn along with a nasopharyngeal swab for COVID-19 infection. The patient was treated conservatively and was injected with midazolam. After his condition got stable, he was transferred to the inpatient department. At that time, the differential diagnoses were stroke, encephalitis, toxic ingestion, or acute onset delirium.

On day two of admission, all the laboratory investigations came out normal. COVID-19 Reverse transcription-Polymerase chain reaction (RT-PCR) test was also negative. His white blood cell count was 7400/μL, platelet count was 264,000/μL, and serum creatinine was 0.8 mg/dL. Serum electrolytes revealed a sodium level of 138 mmol/L, potassium of 4.2 mmol/L, chloride of 101 mmol/L, and bicarbonate of 24 mmol/L. The toxicology screen was normal for common toxins and abusive drugs. On the basis of clinical and laboratory investigations, stroke, encephalitis, and toxic ingestion were excluded. Blood tests for the levels of linezolid were ordered. The level of linezolid was 1600mg/day, which is way more than the toxic dose of 1200mg/day on which 90% of patients can develop side effects. Brief psychotic disorder along with hallucinations was diagnosed on the basis of criteria given by the fifth edition of the Diagnostic and Statistical Manual of Mental Disorders (DSM-5). Linezolid was stopped, and the Naranjo probability score was used to establish a causal relationship between the development of the neuropsychiatric symptoms and linezolid exposure. Our patient's Naranjo probability score for adverse drug reactions (ADRs) was +8. Patient's symptoms began to disappear, and he got stable after two days. He was discharged after physical and psychological counseling. He was followed up in the outpatient department after one week. No symptoms were reported at that time.

## 3 Discussion

Existing literature has reported a prevalence of 0.3–3.8% of psychosis in the patients being treated with antibiotics [3]. Many antibiotics belonging to different classes have been shown to cause both psychosis and new-onset hallucinations. We reported a case of a patient who developed both psychosis and auditory/visual hallucinations induced by toxic blood levels of linezolid.

Linezolid belongs to a newer class of antibiotics that inhibits protein synthesis by preventing the formation of the ribosome complex that begins protein synthesis. It does not exhibit the phenomenon of cross-resistance due to its unique binding site on ribosomal RNA. Therefore, linezolid is being increasingly used for the treatment of multidrug-resistant strains of gram positive bacteria [6]. Linezolid is generally well tolerated with few described side effects.

Serious adverse reactions demanding withdrawal of the drug include lactic acidosis, serotonin syndrome, myelosuppression, and peripheral and optic neuropathy [7]. The safe dosage of linezolid treatment has been established for use only for up to 28 days [8].

The underlying pathophysiology of associations between antibiotics and psychosis remains unclear and may vary by antibiotic class. A study reported by Palaniappan hypothesized that a complex interaction between 5-hydroxytryptamine (5-HT) and dopamine could lead to such kind of neuropsychiatric manifestations [9].

We did a thorough search on different medical databases, including Pubmed and google scholar using the Boolean operator strategy of "((psychosis OR hallucination) AND (neuropsychiatric)) AND (linezolid)." Only one case was identified, which reported linezolid-induced visual hallucinations only [7]. We suggest that further studies should be done to understand this association between prolonged linezolid exposure and atypical neuropsychiatric symptoms, along with a better insight into underlying pathophysiology. Physicians, especially infectious disease specialists, should monitor the blood levels of antibiotics for optimal management of the patients.

## 4 Conclusion

On rare occasions, toxic blood levels of linezolid can lead to neuropsychiatric manifestations. Both linezolid-induced psychosis and hallucinations are manageable by suspension of the drug. Physicians should monitor the blood levels of this antibiotic to keep their patients safe from such serious adverse effects.

## Ethics approval and consent to participate

Written informed consent for participation and publication was sought from the patient as per hospital protocol. Ethical approval was also obtained from the institutional review board.

## Consent for publication

Written informed consent was obtained from the patient for publication of this case report and any accompanying images. Informed consent was also taken from the patient's kin, his mother in this case. A copy of the written consent is available for review by the Editor-in-Chief of this journal.

## Guarantor

Dr Hamza Maqsood, Nishtar Medical University And Hospital, Multan, Pakistan Phone: +923168435531 Email address: hamzamaqsood381@gmail.com.

## Consent

Written informed consent was obtained from the patient for publication of this case report and accompanying images. A copy of the written consent is available for review by the Editor-in-Chief of this journal on request.

## Availability of data and materials

Not applicable.

## Funding

This study received no external funding.

## Registration of research studies

1. Name of the registry: NA.

2. Unique Identifying number or registration ID: NA.

3. Hyperlink to your specific registration (must be publicly accessible and will be checked): NA.

## Provenance and peer review

Not commissioned, externally peer reviewed.

## Authors contributions

HM conceived and designed the study. SS and SA were responsible for data collection and acquisition of data. HM, SS, SA, and SY analyzed and/or interpreted the data. HM and SY performed the literature review. HM, SS, and SA wrote the initial manuscript. SY and SN critically revised the manuscript. All authors have approved the final manuscript.

## Declaration of competing interest

None declared.
